# Supplementary material for: A minus-end directed kinesin motor directs gravitropism in Physcomitrella patens
Source: Nat Commun. 2021 Jul 22;12:4470. doi: 10.1038/s41467-021-24546-2 (PMC8298521; doi:10.1038/s41467-021-24546-2)
Supplement: Supplementary file 1 — Supplementary Information [file 41467_2021_24546_MOESM1_ESM.pdf]

Supplementary Figures

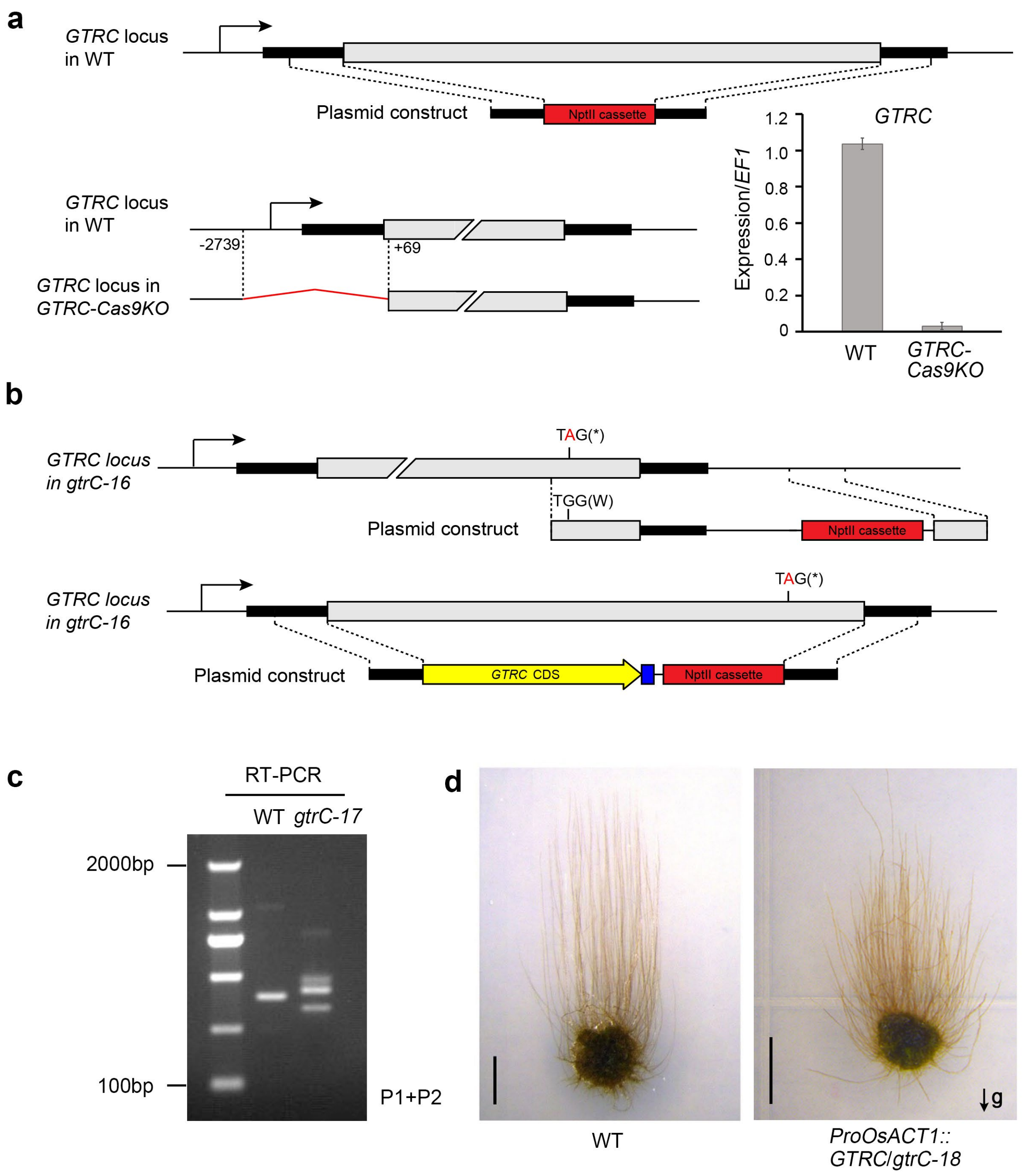

Supplementary Figure 1. Constructs and genotypes for genetic confirmation of *GTRC*. a, The whole *GTRC* genomic sequence was substituted with the NptII cassette via homologous recombination, generating *GTRC-HRKO*. A sgRNA was designed to target the first exon of *GTRC*, resulting in deletion of a large fragment of the *GTRC* promoter, and the mutant line was named *GTRC-Cas9KO*, in which *GTRC* is not expressed. The red line indicates the deleted fragment. **b**, *HARes/gtrC-16* was generated by replacing the mutated nucleotide in the *GTRC*

gene with the wild type nucleotide via homologous recombination. *ProGTRC::GTRC/gtrC-16* was generated by substituting the *GTRC* genomic sequence in *gtrC-16* with the *GTRC* coding sequence. Gray box: *GTRC* genomic region. Black box: Untranslated region (UTR). Red box: NptII cassette. Yellow box: *GTRC* coding sequence (CDS). Blue box: Transcription terminator. **c**, Splicing is altered in *gtrC-17*. RNA was extracted from WT and *gtrC-17,* and reverse-transcribed into cDNA. Primers P1 and P2 were designed to examine the intron splicing, the positions of which are shown in the gene structure (Fig. 2c). The experiment was carried out once. Source data are provided as a Source Data file. **d**, Complementation of *gtrC-18*. The GTRC coding sequence driven by the OsActin promoter was used to transform *gtrC-18,* and then the protonemata were grown in the dark on vertically oriented plates. In **d**, the arrows with "g" show the direction of gravity. Scale bars, 3 mm.

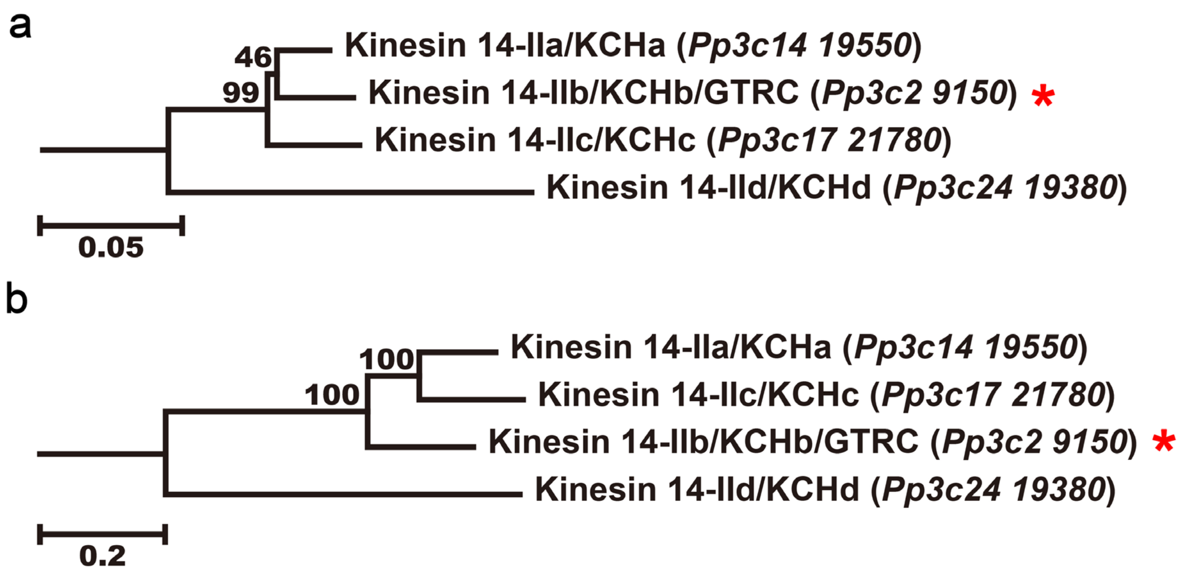

Supplementary Figure 2. KCH family members in *P. patens*. Phylogenetic tree of four *P. patens* KCH members based on motor domain (**a**) or full-length protein sequence (**b**). Red asterisks highlight GTRC. Bars indicate the amino acid substitutions per site.

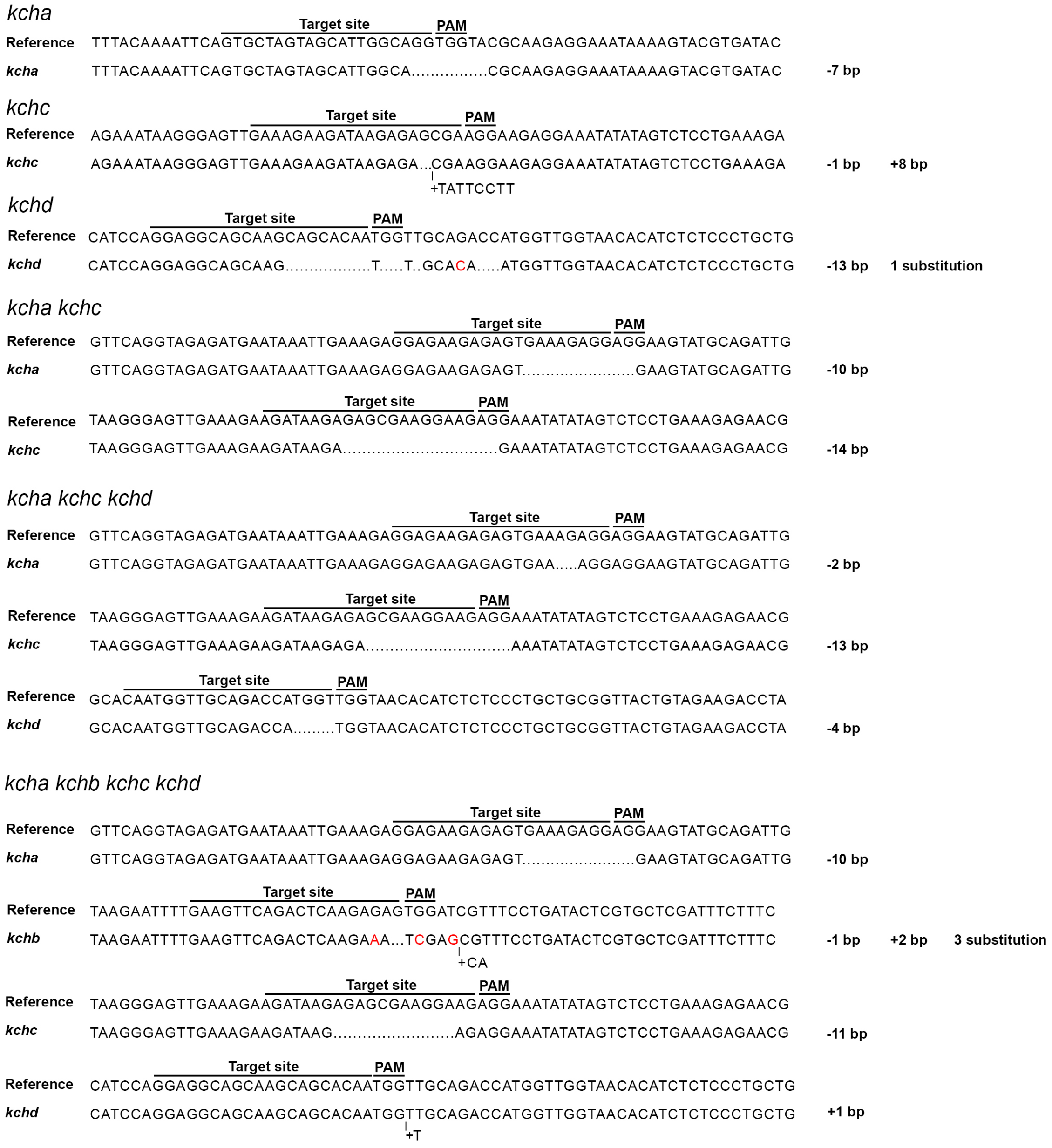

Supplementary Figure 3. Genotypes of CRISPR knockout lines of *KCH* subfamily genes. For each *KCH* gene, one sgRNA was designed to edit the genome. Deletions were symbolled as dots. Insertions were added by "+" at the certain base pair. Substituted base pairs were marked by red. The target site along with PAM sequence were underlined.

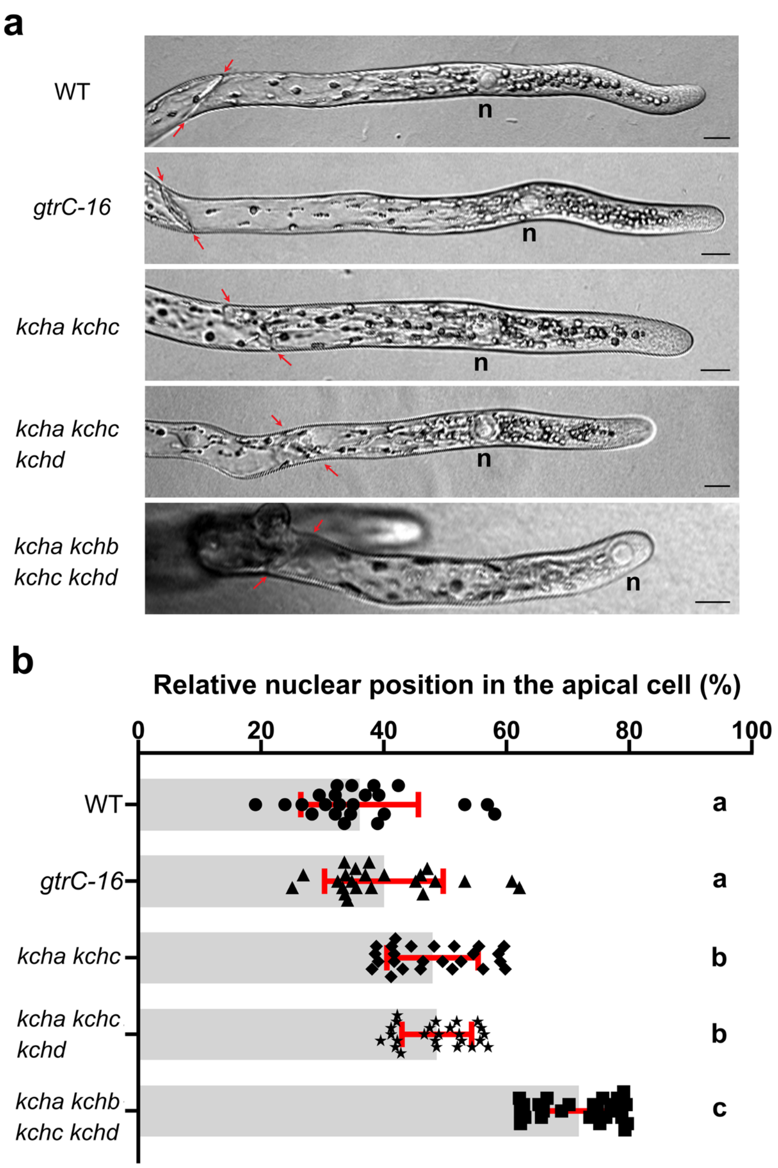

Supplementary Figure 4. Nuclear positioning in *kch* mutants. a, Distribution of nuclei in 7-d-old protonemal tip cells. Red arrows and "n" indicate the positions of the cell wall and nucleus, respectively. Bars = 10 μm. The images of more than 20 tip cells for each genotype were collected, and one representative for each genotype is shown. **b,** Relative position of the nuclei within the apical cells were quantified. The "0" corresponds to the cell wall between apical and second cells, whereas "100" indicates the cell tip. Bars and error bars represent the mean and sd, respectively. WT, n = 23; *gtrC-16*, n = 23; *kcha kchc*, n = 25; *kcha kchc kchd*, n = 25; *kcha kchb kchc kchd*, n = 25. Letters a, b, c indicate significant differences, analyzed by One-way ANOVA and Games-Howell Post-hoc Tests. $P<0.05$. Source data are provided as a Source Data file. Experiments and data analyses were performed twice, with data from one experiment displayed.

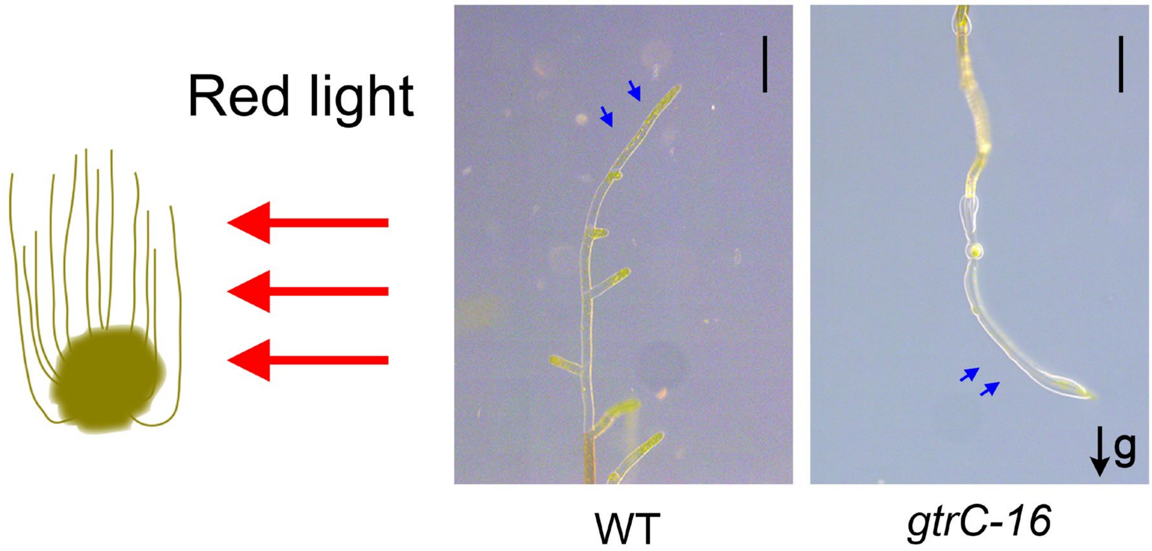

Supplementary Figure 5. Mutation of *GTRC* has no effect on phototropism. Wild type *P. patens* and representative mutants of *GTRC* was grown on vertically-oriented culture plates for around 1 week in the dark and then transferred into unilateral red light (5 μmol/m^2·s). Blue arrows indicate where protonemata bent toward red light. Scale bars, 1mm.

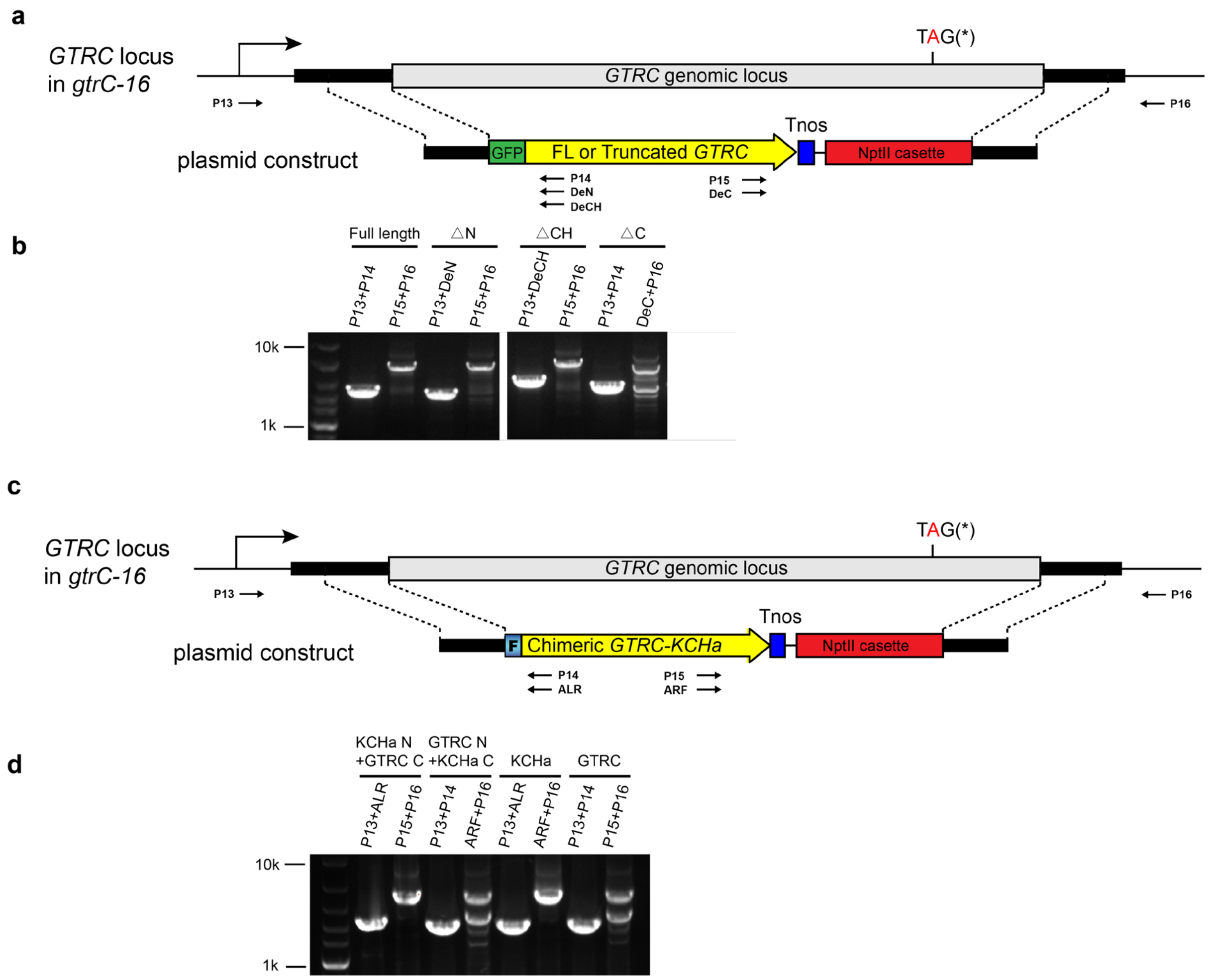

Supplementary Figure 6. Constructs for revealing the localization and domain function of GTRC. a, To generate lines expressing GFP fused with full length (FL) or truncated GTRC in *gtrC-16*, *GTRC* genomic sequence in *gtrC-16* was substituted by various constructs of *GFP* fused truncated *GTRC* via homologous recombination. **b,** Agarose-gel electrophoresis analysis of targeted lines. The primers are labelled in panel a. **c,** To generate lines expressing GTRC, KCHa and chimeric protein between GTRC and KCHa, the corresponding constructs N-terminally fused with 3×*FLAG* (F) were transformed to substitute *GTRC* genomic sequence in *gtrC-16*. **d,** Agarose-gel electrophoresis analysis of targeted lines. The primers are labelled in panel c. Source data are provided as a Source Data file. Agarose-gel electrophoresis analyses in b and d were repeated twice with similar results.

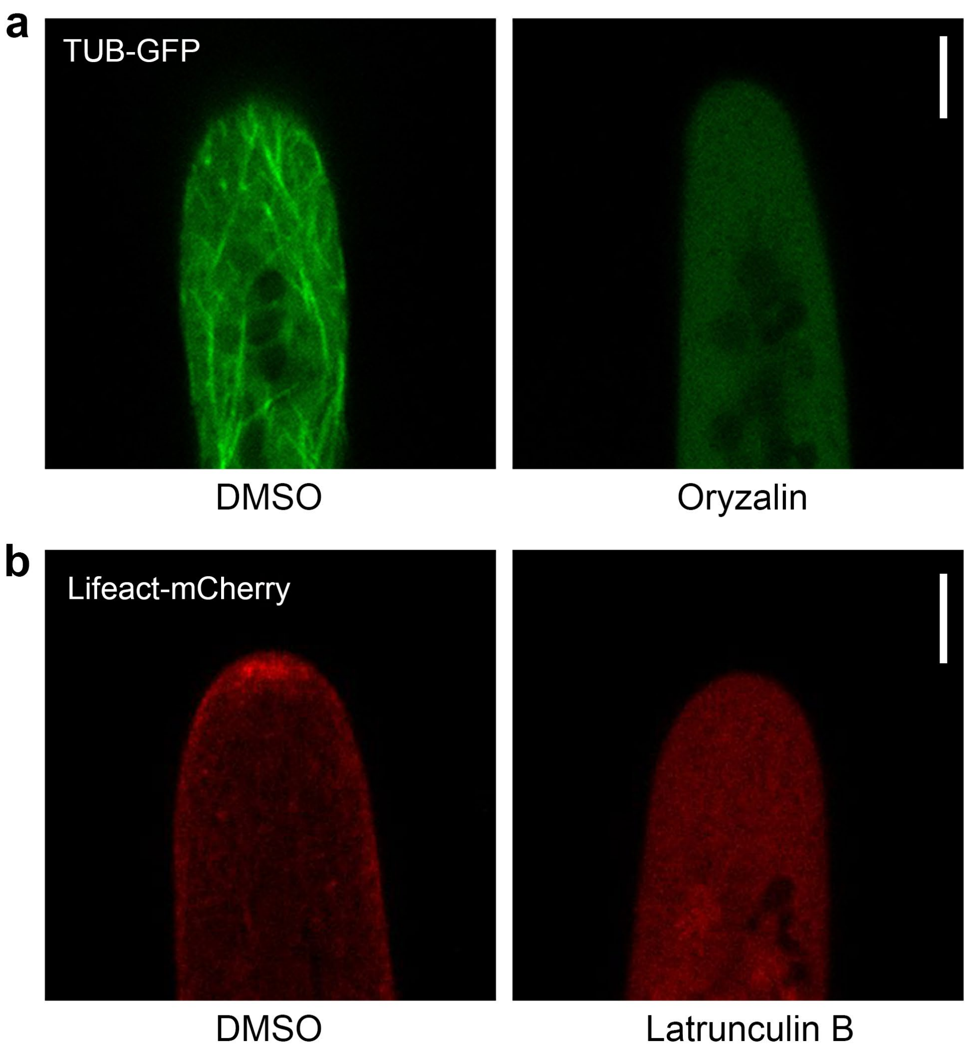

Supplementary Figure 7. Treatments of wild type *P. patens* protonemata with Oryzalin and Latrunculin B. a, Microtubule was severely disrupted after oryzalin (25 μM) treatment for 10 minutes. The fluorescence of 13 tip cells treated with Oryzalin displayed similar results, and a representative is shown. **b,** Actin cluster disappears after Latrunculin B (50 μM) treatment for 10 minutes. The fluorescence of 8 tip cells treated with Latrunculin B displayed similar results, and a representative is shown. In both a and b, the final concentration of DMSO was < 0.1% (v/v). Scale bars, 5 μm.

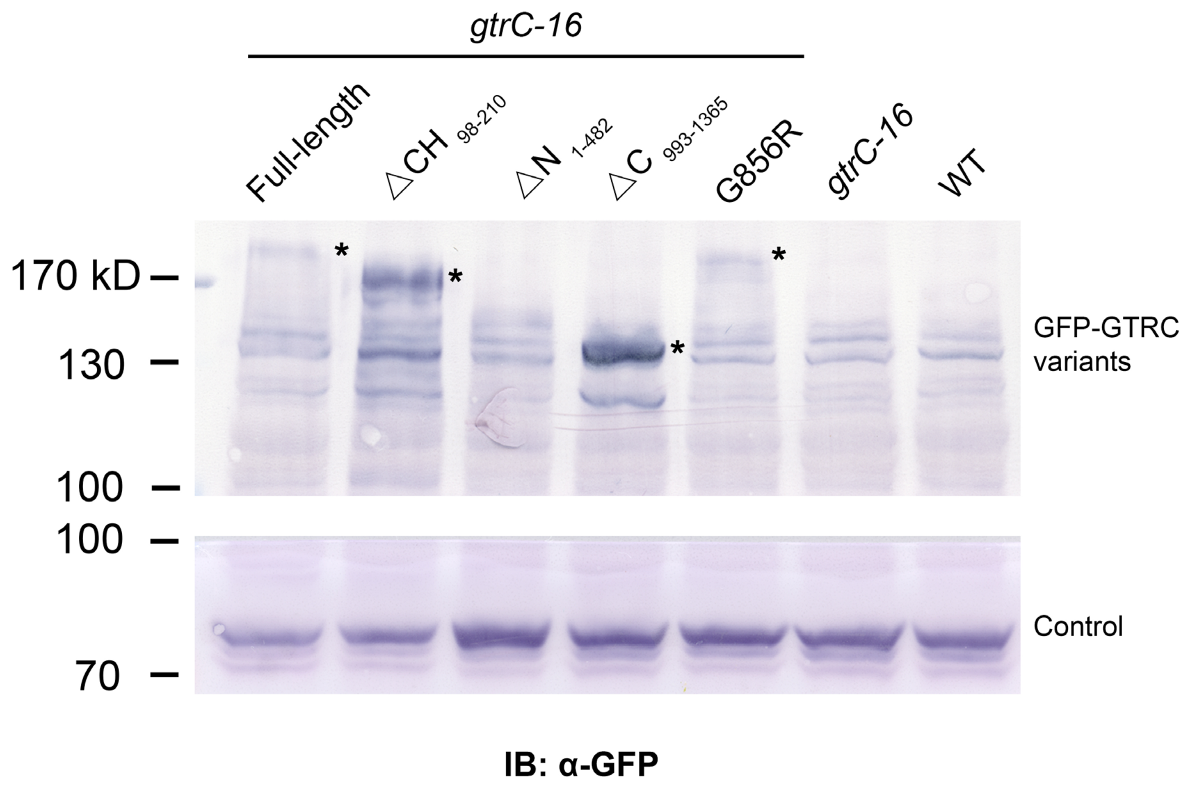

Supplementary Figure 8. Expression analysis of full length or truncated GFP-GTRC proteins. Total proteins were extracted from 7-d-old dark-grown protonema tissue by RIPA buffer with protease inhibitor and analyzed by Western blot using anti-GFP antibody. The unspecific band between 70 kD and 100 kD was used as a loading control. Asterisks indicate the full length or truncated GFP-GTRC proteins. Source data are provided as a Source Data file. The experiments were repeated twice with similar results.

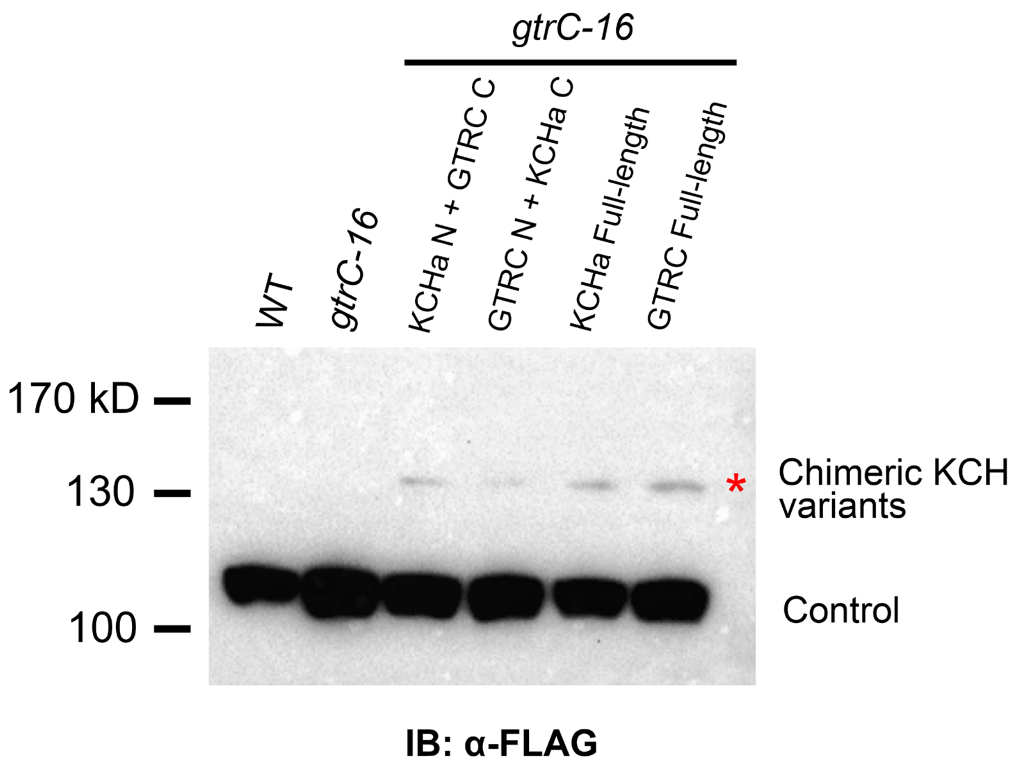

Supplementary Figure 9. Expression analysis of GTRC, KCHa or chimeric KCH proteins. Total proteins were extracted from 7-d-old protonema tissue using RIPA buffer and analyzed by Western blot using anti-FLAG antibody. The asterisk indicates the position of the full length GTRC, KCHa or chimeric KCH proteins fused with FLAG. An unspecific band between 100 kD and 130 kD was used as a loading control. Source data are provided as a Source Data file. The experiments were repeated twice with similar results.

a

	Up	Down
Lifeact-mCherry	10	0
Lifeact-mCherry/*gtrC*	3	7

b

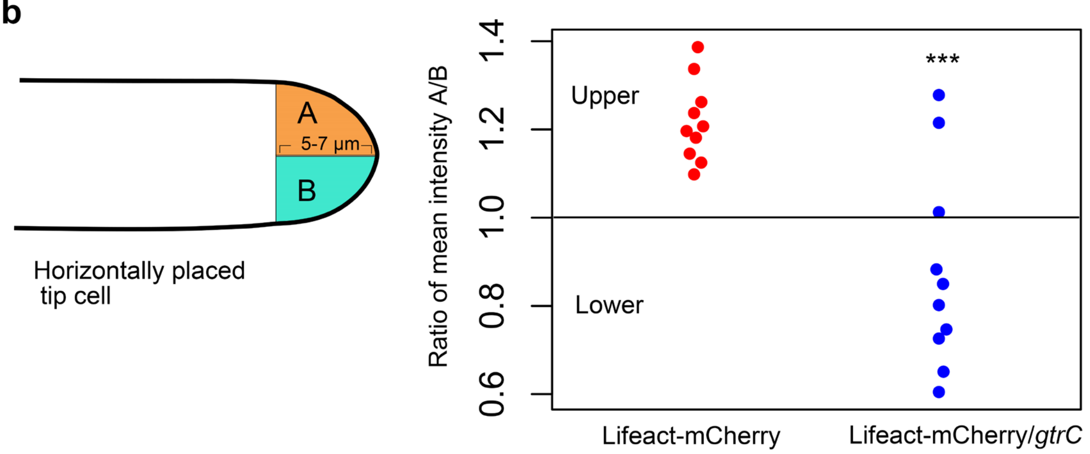

Supplementary Figure 10. Statistics of actin distribution in protonema tip cells during gravistimulation. a, Numbers of gravistimulated dark-grown protonemata expressing Lifeact-mCherry. Up, the protonema tip cell bends upwards; Down, the protonema tip cell bends downwards. The up or down was judged when the tip cells showed first obvious bending. Some of the first bending of Lifeact-mCherry/*gtrC* protonemata were upward due to their wavy growth, which would eventually grow downwards if they kept growing for enough time, as demonstrated in Figures 1b, 1c, 6a and 6b. The protonemata growing horizontally might represent the cells losing gravitropic response due to laser effect, which were excluded in the statistics. **b,** Left, schematic diagram of horizontally placed tip cell under gravistimulation. A 5-7 μm apical region of each tip cell was divided into an upper half (A) and a lower half (B), and ratios of their mean fluorescence intensity were calculated ahead of the first obvious bending. Right, statistical analysis of actin fluorescence intensity distribution. The ratios were determined as significantly different between wild type and *gtrC* by a two-sample t-test (***P < 0.001). Source data are provided as a Source Data file.

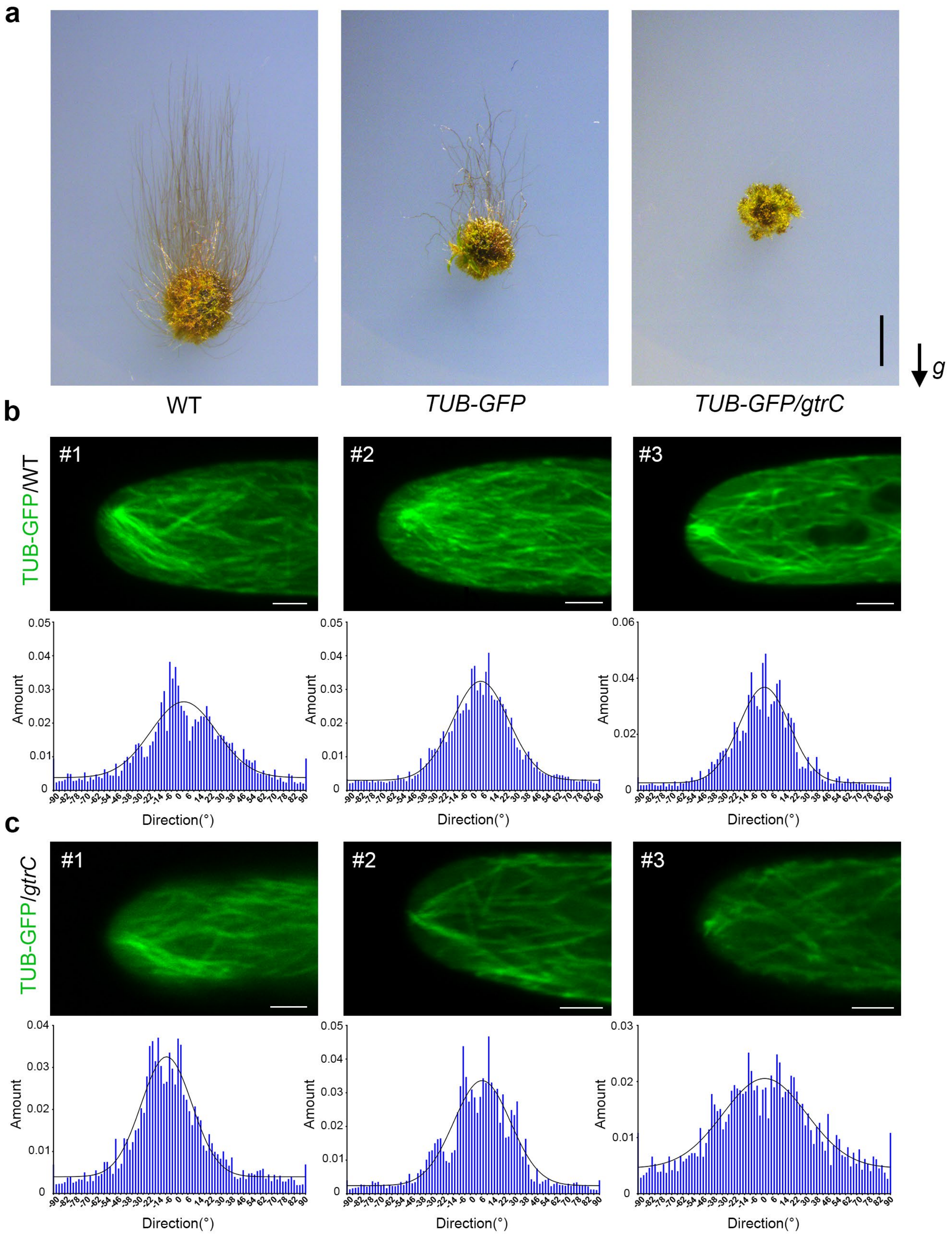

Supplementary Figure 11. Microtubule orientations in *GTRC* knock out lines are similar to wild type (WT). a, Wild type Gransden (Gd) strains of *P. patens, TUB-GFP*, and *TUB-GFP/gtrC* lines were grown for around 2 weeks vertically in the dark. Scale bar, 3 mm. **b,** Top, fluorescence of TUB-GFP was collected in three representative protonema tip cells grown in the dark for 7 days. Bottom, microtubule orientations of these cells are quantified using the

ImageJ plugin Directionality. Calculation method was set as Fourier components. The histograms represent the relative frequency of the structures (microtubules) in given orientations within an image. The total number of microtubules calculated in the histogram is around 100 to 300 for each tip cell. **c,** *GTRC* was knocked out in the TUB-GFP line to generate TUB-GFP/*gtrC*, and microtubule orientations in the protonema tip cells were quantified as in wild type. In b and c, around 10 cells were observed for each genotype. Scale bars, 5 μm.

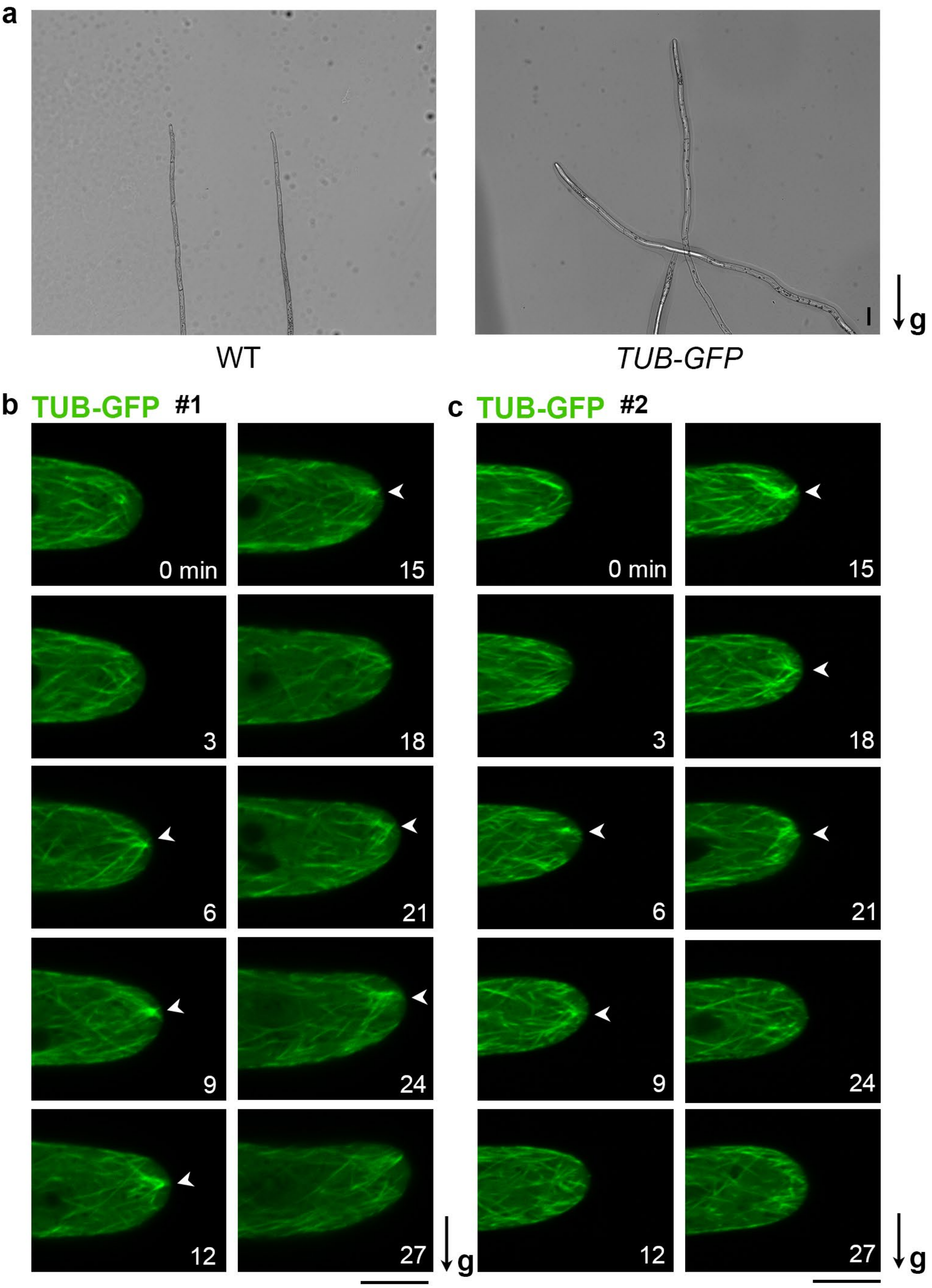

Supplementary Figure 12. The distribution of microtubules during gravistimulation. a, The tips of *TUB-GFP* protonemata were curled when they were grown on vertical plates in the dark, which showed poor gravitropic responses due to the insertion of TUB-GFP. Scale bar, 50

μm. **b, c,** Protonemata expressing TUB-GFP were grown in the dark vertically for 7 days and then turned 90 degrees for gravistimulation. Images were captured every 3 minutes, and two representative tip cells were shown. The tip cell #1 showed obvious negative gravitropism within half an hour, and tubulin foci (indicated by white arrowhead) located on the upper side of apical region ahead of bending (**b**). The tip cell #2 didn't showed obvious negative gravitropism within half an hour, and the tubulin foci located randomly at the upper or lower side of apical region (**c**). Due to the TUB-GFP insertion and laser effect during observation, most of the protonemata (18 out of 20) did not show obvious negative gravitropism, and their growth and fluorescence pattern were similar to tip cell #2. For the two protonemata showing negative gravitropism, both of them showed asymmetric fluorescence pattern as tip cell #1. Arrows labelled with "g" indicate the direction of the gravity vector. Scale bars, 10 μm.

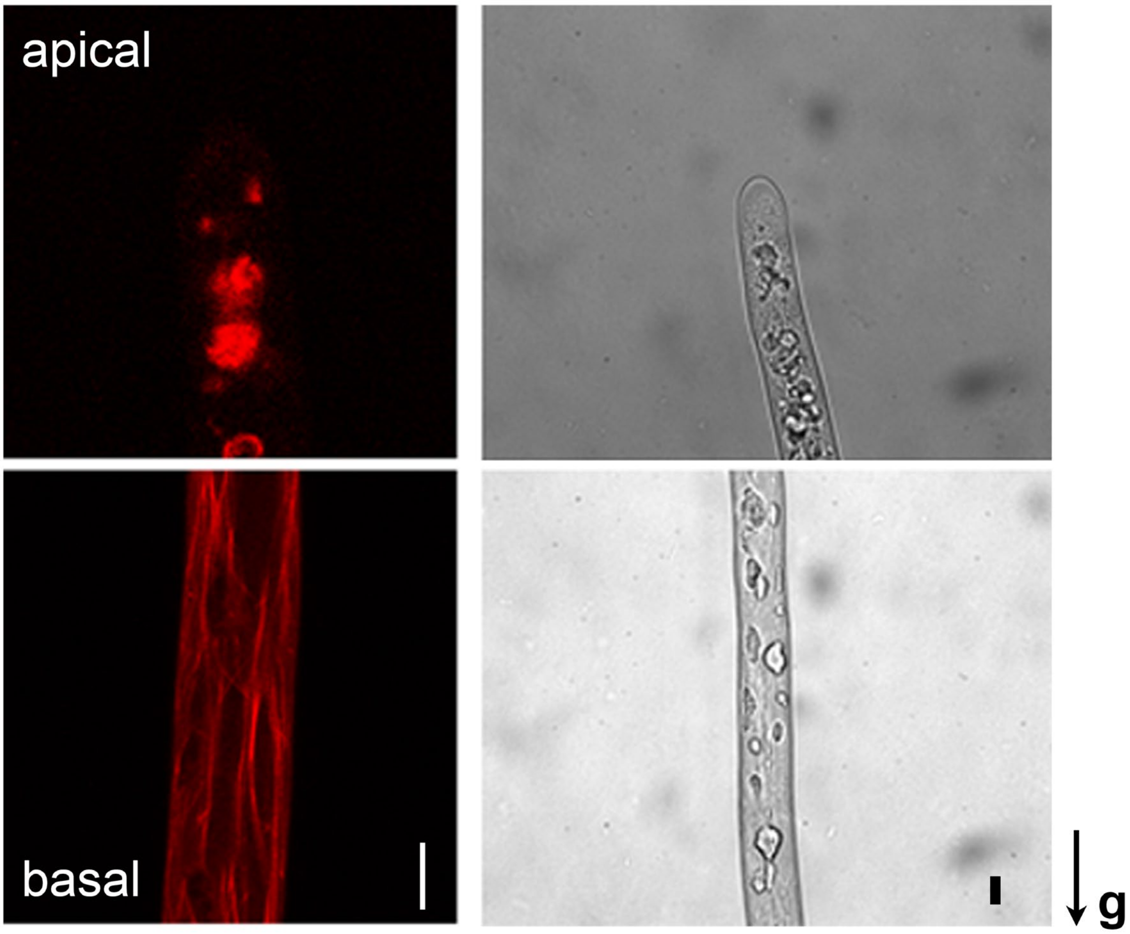

Supplementary Figure 13. Microtubule signal intensity in the protonema transformed with p7113-mRFP-tub. The RFP fluorescence in the apical and basal parts of dark-grown protonema was collected by a ZEISS LSM800 confocal microscope. The corresponding bright field view is shown on the right. The arrow labelled with “g” indicates the direction of gravity. Scale bar, 5 μm. The fluorescence of four protonemata were collected, and the results were similar.

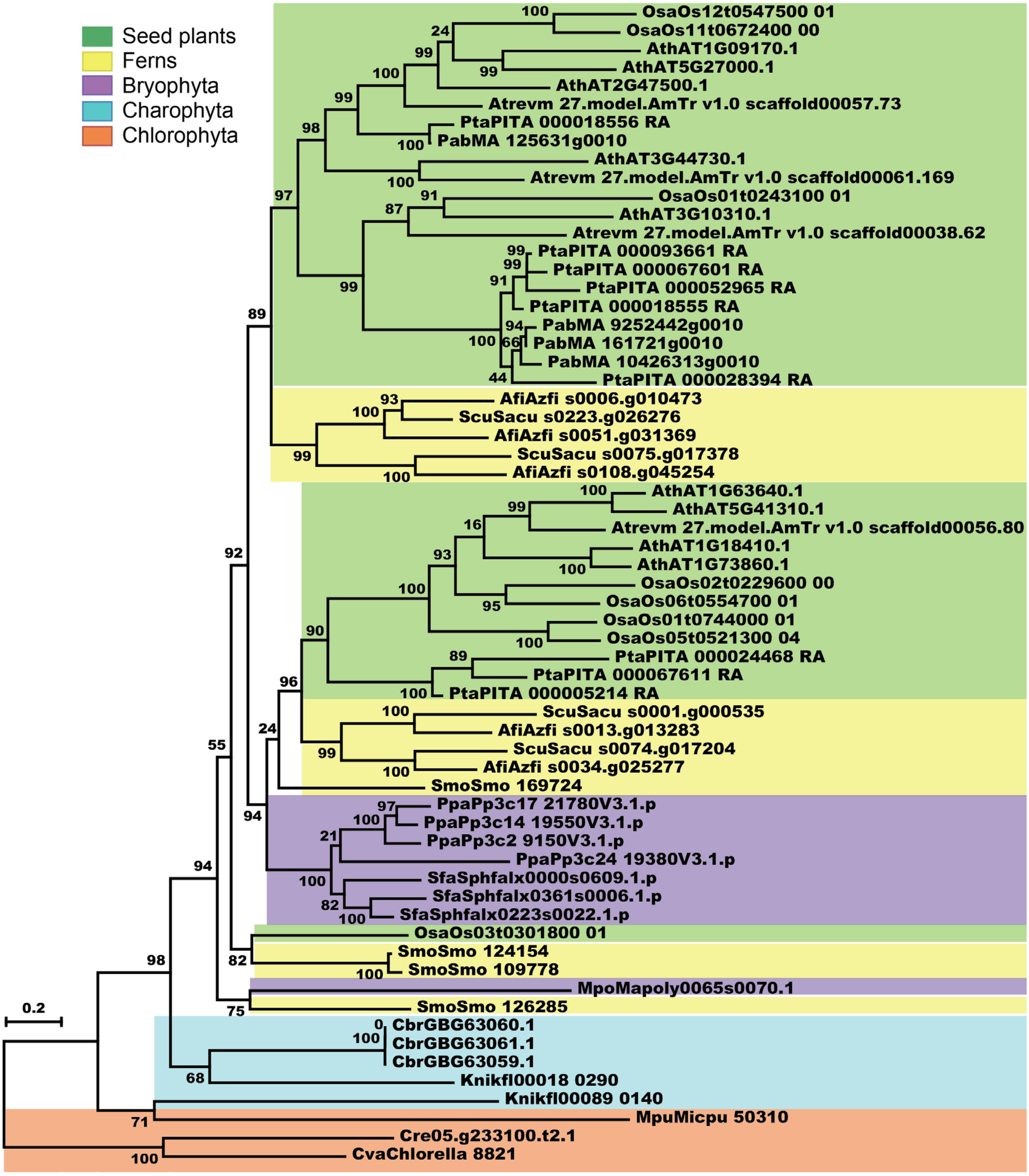

Supplementary Figure 14. Phylogenetic tree of *GTRC* homologs (*KCH*) in multiple organisms. Phylogenetic tree based on full length protein sequences. The scale bar indicates amino acid substitutions per site. Numbers on the nodes show the bootstrap percentage. Mpo: *Marchantia polymorpha*; Ppa: *Physcomitrella patens*; Sfa: *Sphagnum fallax*; Smo: *Selaginella moellendorffii*; Afi: *Azolla filiculoides*; Scu: *Salvinia cucullata*; Pab: *Picea abies*; Atr: *Amborella trichopoda*; Ath: *Arabidopsis thaliana*; Osa: *Oryza sativa*; Mpu: *Micromonas pusilla*; Cre: *Chlamydomonas reinhardtii*; Cva: *Chlorella variabilis*; Kni: *Klebsormidium nitens*; Cbr: *Chara braunii*; Pta: *Pinus taeda*.

Supplementary Table

Supplementary Table 1. Primers used in this study

CRISPR system for *P. patens* construct	PIG1b L_XbaI F	GCTCTAGAGTGTATTCTATTTGATTGATAAG
	PIG1b L_SacI R	CGAGCTCAAACACATTTTTATGTGGGCCG
	PIG1b R_KpnI F	GGGGTACCTAAGATTTCTATGCACGGATAGC
	PIG1b R_HindIII R	GGAATTCGCGGCCGCAAGCTTAAAACATGAATAACC AAATTAAA
	35S_HindIII F	CCCAAGCTTAGATTAGCCTTTTCAATTTCAGA
	Tnos_NotI R	ATAGTTTAGCGGCCGCCCGATCTAGTAACATAGATGA CA
	pBS-Modified F	GGGGAGAGGCGGTTTGCGTATTGGGCGCTCAACCGC TTCCTCGCTCACTGACTCGCTGC
	pBS-Modified R	GCAGCGAGTCAGTGAGCGAGGAAGCGGTTGAGCGCC CAATACGCAAACCGCCTCTCCCC
	PpU6 F	GGactagtATTGAATGTCCATTGAAGCAGAC
	PpU6 R	AAACAGGTCTTCTCGAAGACCCGGTTGTAAGTCCTCC ACCTTCCT
	PpU6_KpnI F	GGGGTACCactagtATTGAATGTCCATTGAAGCA
	Scaffold_SacI R	CGAGCTCAAGCTTATCGCTAGCCTAAAAAAAAGCAC CGACTCG GTGCCACTTTTTC
	SapI F	AACCGAAGAGCGCGGCCGCCTCGAGGCTCTTCt
	SapI R	AAACAGAAGAGCCTCGAGGCGGCCGCGCTCTTC
	ccdB_NotI F	ATTTGCGGCCGCCTGGCTTATCGAAATTAATACGACT
	ccdB_XhoI R	CCGCTCGAGTTCGAACCGCGGGCCCTCTAGATCAACC
	HPT_HindIII F	CCCaagcttCCCCTACTCCAAAAATGTCAAAG
	HPT_SacI R	CGAGCTCTAGTTTTGATCTTGAAAGATCTT
	PpKCHb-sgRNA1-F	AACCGGAGCGATGCCTCATGGCAT
	PpKCHb-sgRNA1-R	AACATGCCATGAGGCATCGCTCCG
	PpKCHb-sgRNA2-F	AACCGAAGTTCAGACTCAAGAGAG
	PpKCHb-sgRNA2-R	AACCTCTCTTGAGTCTGAACTTCG
	IIa-sgRNA-F	AACCGGAGAAGAGAGTGAAAGAGG
	IIa-sgRNA-R	AACCCTCTTTCACTCTCTTCTCC
	IIc-sgRNA-F	AACCGAAAGAAGATAAGAGAGCGA
	IIc-sgRNA-R	AACTCGCTCTCTTATCTTCTTTC
	IId-sgRNA-F	AACCGAGAGATGTGTTACCAACCA
	IId-sgRNA-R	AACTGGTTGGTAACACATCTCTC
Knockout of *GTRC* by homologous recombination	FE2KO-LF1	GGGGTACCGGGTGCTGAGGAACTGATTTGG
	FE2KO-LR1	CCATCGATGAATTCCCGATCTAGTAACATAG
	FE2KO-RF1	GCTCTAGAAGGGTAATTTCCTTGTATGGC
	FE2KO-RR1	CGGAGCTCTACGAAGTTAATTTGCATAA

Genetic complementation and subcellular localization	csKCH_RF	TCTAGAAGGGTAATTTCCTTGTATGG
	csKCH_RR	GAGCTCTACGAAGTTAATTTGCATAAAAATG
	csKCH_LF1	GGGGTACCGGGTGCTGAGGAACTGATTTGG
	csKCH_LFIR	GCTAGCAGGCCTGCGGCCGCAACTACGGCGTTCAAACAAAATTAC
	csKCH_LFIF	GCGGCCGCAGGCCTGCTAGCGGATCCACTAGTGAGCTC
	csKCH_LR1	CCATCGATGAATTCCCGATCTAGTAACATAG
	GTRCcdsF	GCGGCCGCATGGATGTGGCTAGAATGGGTTATG
	GTRCcdsR	GCTAGCCTATCTCCAGGACGTTGAGGAGC
	GFP-GTRCF	GCGGCCGCATGGTGAGCAAGGGCGAGGAGC
	GFP-GTRCIR	CCTCCACCGCCTTCTTCTATTCCTTCGGAACTACCCTTGTACAGCTCGTCCATGCCGTG
	GFP-GTRCIF	ATAGAAGAAGGCGGTGGAGGGTGCGGTGGTGGTGGTTGTATGGATGTGGCTAGAATGGG
	GFP-GTRCR	GCTAGCCTATCTCCAGGACGTTGAGGAGC
	GD-deCH_IR	ACCGCCCTGCTTCCAGTCATATGCTGATTCCTCTGCCCTTC
	GD-deCH_IF	GAAGGGCAGAGGAATCAGCATATGACTGGAAGCAGGGCGGT
	GD-deN_IR	CATTCTAGCCACATCCATGACGAAGCGACGACTCGATC
	GD-deN_IF	GATCGAGTCGTCGCTTCGTCATGGATGTGGCTAGAATG
	GD-deC_R	GCTAGCCTATGAGAGTTCAGCATCTTTCTTAG
	TrbcS-F	GCTCTAGAAGCTTTCGTCCGTATCATCG
	TrbcS-R	TCCCCCGGGCAAAAAGCCTATACTGTACT
	OsACT1-F	CCGCGGCCTCAAGCTTCGAGGTCA
	OsACT1-R	GCGGCCGCTTCTACCTACAAAAAAGCTC
	aCbN_F	AAGGAAAAAAGCGGCCGCATGGATGTGGCTAGAATGGGTTATGAG
	aCbN_IR	CTTCAAGATCCTGGACTTATACTTCTCCACCCCGTTCCCTG
	aCbN_IF	CAGGGAACGGGGTGGAGAAGTATAAGTCCAGGATCTTGAAG
	aCbN_R	CTAGCTAGCTTACCTCCAAGAGGTTGAGGAC
	aNbC_F	AAGGAAAAAAGCGGCCGCATGGATGTGGCAAGAATGGGG
	aNbC_IR	GTTGTCCTGGACTTAAACTTCTCCACGCCAACAC
	aNbC_IF	GTGTTGGCGTGGAGAAGTTTAAGTCCAGGACAAC
	aNbC_R	CTAGCTAGCCTATCTCCAGGACGTTGAGG
	KCHaF	AAGGAAAAAAGCGGCCGCATGGATGTGGCAAGAATGGGG
	KCHaR	CTAGCTAGCTTACCTCCAAGAGGTTGAGGAC

	GTRC_F	AAGGAAAAAAGCGGCCGCATGGATGTGGCTAGAATG GGTTATGAG
	GTRC_R	CTAGCTAGCCTATCTCCAGGACGTTGAGG
	LarmF	GGGGTACCGGGTGCTGAGGAACTGATTTGG
	3F-IR	CTTTGTAATCAATATCATGATCCTTGTAGTCTCCGTCG TGGTCCTTATAGTCCATAACTACGGCGTTCAAAC
	3F-IF	GACTACAAGGATCATGATATTGATTACAAAGACGAT GACGATAAGATGGTGAGCAAGGGCGAGG
	3F-notR	AAGGAAAAAAGCGGCCGCAACAACCACCACCACCGC AC
Construction of gene-targeted *gtrC-5* $Q^{598}X$ mutant in WT (Gd)	KHC_S6	CCTCCTTGTATCCGAACAGATTG
	KHC_WT_A	ATGCTGCTCGAGCCATACTCTG
	KHC_mWT_A	ATGCTGCTCGAGCCATTGATTG
	KHC_mut_A	ATGCTGCTCGAGCCATTGATTA
	KHC_mutS	TGCTTGTGCAGAAGAACAGCTTTAATCAATGGCTCGA GCAGCATCTGG
	KHC_A7	TCGATTCAACACATTCCAGACAA
	KHC_KIS	ATGCGGTTCATCCTTCATAATGT
	KHC_KIA	CCAACGGAGTTCATTGTAAGTCG
Primers for SNP and SSR mapping of *gtrC-5* interval in segregants	SNP_15286FG	GTCATCCACTTGAAAATATGGTTTG
	SNP_15286FV	GTCATCCACTTGAAAATATGGTTTA
	SNP_15286R	ACCTCAAGAAGCGAGGTCAA
	Sc237_511583FG	GCTTTACTCACCAGGATGTCAC
	Sc237_511583FV	GCTTTACTCACCAGGATGTCAA
	Sc237_511583R	ATGAGGCACATTTGATGCAG
	SNP_15299FG	GATTATTTGGAGGCTGGCAC
	SNP_15299FV	GATTATTTGGAGGCTGGCAG
	SNP_15299R	AATCGACGCCATTAGCAAAC
	SNP_8641FG	GAGCAGCTTGGATAGACACAG
	SNP_8641FV	GAGCAGCTTGGATAGACACAA
	SNP_8641R	TATTTGGCGCTTCTGGTATG
	Sc84_563431FG	TGCAGCTAGGTAGTAGCGCG
	Sc84_563431FV	TGCAGCTAGGTAGTAGCGCA
	Sc84_563431R	CACCCTCGACAATCCTCAAC
	SSR_723F	GTTGCACGCAGAAGCTGATA
	SSR_723R	TCCTTTCGTCCCACAGTAGC

Primers for Genotyping	P13	TGATGATGATGATGCGGGGA
	P14	GAGAGATTCCGTTCCTCAGAC
	P15	TGGGCAGTTGAAAATCAAG
	P16	CCGATTCCCAGCAGACGATA
	DeN	GTGTCGAGTTGGGACAACCT
	DeC	TAGCTGCATTGGCACAAAAG
	DeCH	ACTTCATAGATGGGCCGTTG
	ALR	CGTACTCCTGCCGAAGAGAC
	ARF	GGCATGTGCCTTACAGGAAT
