# Supplementary material for: A minus-end directed kinesin motor directs gravitropism in Physcomitrella patens
Source: Nat Commun. 2021 Jul 22;12:4470. doi: 10.1038/s41467-021-24546-2 (PMC8298521; doi:10.1038/s41467-021-24546-2)
Supplement: Supplementary file 3 — Descriptions of Additional Supplementary Files [file 41467_2021_24546_MOESM3_ESM.pdf]

Descriptions of Additional Supplementary Files

Supplementary Movie 1

Description: Movement of GFP-GTRC in the dark-grown protonema of GFP-GTRC line. Protonemal cells of GFP-GTRC line were placed onto glass-bottom plates coated with a thin layer of BCDAT medium, and cultured at 25 ^{o}C under continuous white light for around 4 days and then moved into darkness for around a week. Time-lapse images were recorded in tip cells with a spinning disk confocal microscope (ZEISS, CSU-X1) with an interval of 250 ms. Red arrow indicates the movement of GFP-GTRC.

Supplementary Movie 2

Description: Movement of GFP-GTRC on the microtubule in the light-grown protonema of GFP-GTRC/RFP-TUB line. Protonemal cells of GFP-GTRC/RFP-TUB line was placed onto glass-bottom plates coated with a thin layer of BCDAT medium, and cultured at 25 ^{o}C under continuous white light for around 4 days. Time-lapse images were recorded with a spinning disk confocal microscope (Andor Dragonfly) with an interval of 630 ms. White arrow indicates the GFP-GTRC moving along MT.
