# Supplementary material for: A minus-end directed kinesin motor directs gravitropism in Physcomitrella patens
Source: Nat Commun. 2021 Jul 22;12:4470. doi: 10.1038/s41467-021-24546-2 (PMC8298521; doi:10.1038/s41467-021-24546-2)
Supplement: Supplementary file 6 — Reporting-summary [file 41467_2021_24546_MOESM6_ESM.pdf]

nature research

Corresponding author(s): Haodong Chen

Last updated by author(s): Jun 10, 2021

Reporting Summary

Nature Research wishes to improve the reproducibility of the work that we publish. This form provides structure for consistency and transparency in reporting. For further information on Nature Research policies, see Authors & Referees and the Editorial Policy Checklist .

Statistics

For all statistical analyses, confirm that the following items are present in the figure legend, table legend, main text, or Methods section.

n/a	Confirmed	
☐	☒	The exact sample size (*n*) for each experimental group/condition, given as a discrete number and unit of measurement
☐	☒	A statement on whether measurements were taken from distinct samples or whether the same sample was measured repeatedly
☐	☒	The statistical test(s) used AND whether they are one- or two-sided<br>*Only common tests should be described solely by name; describe more complex techniques in the Methods section.*
☒	☐	A description of all covariates tested
☒	☐	A description of any assumptions or corrections, such as tests of normality and adjustment for multiple comparisons
☐	☒	A full description of the statistical parameters including central tendency (e.g. means) or other basic estimates (e.g. regression coefficient) AND variation (e.g. standard deviation) or associated estimates of uncertainty (e.g. confidence intervals)
☒	☐	For null hypothesis testing, the test statistic (e.g. *F*, *t*, *r*) with confidence intervals, effect sizes, degrees of freedom and *P* value noted<br>*Give P values as exact values whenever suitable.*
☒	☐	For Bayesian analysis, information on the choice of priors and Markov chain Monte Carlo settings
☒	☐	For hierarchical and complex designs, identification of the appropriate level for tests and full reporting of outcomes
☐	☒	Estimates of effect sizes (e.g. Cohen's *d*, Pearson's *r*), indicating how they were calculated

Our web collection on statistics for biologists contains articles on many of the points above.

Software and code

Policy information about availability of computer code

Data collection	imageJ 1.52n, fastqc v0.11.6, Seqtk-1.3 (r106), GATK 3.7-0-gcfedb67, bcftools v1.6, SAMtools v1.6, bwa-0.7.16a, ANNOVAR
Data analysis	imageJ 1.52n, Coloc 2, R version 3.5.2, InterProScan v5.24, MAFFT, Phyutility program v2.2.6, ProtTest v3.4.2,FastTree v2.1

For manuscripts utilizing custom algorithms or software that are central to the research but not yet described in published literature, software must be made available to editors/reviewers. We strongly encourage code deposition in a community repository (e.g. GitHub). See the Nature Research guidelines for submitting code & software for further information.

Data

Policy information about availability of data

All manuscripts must include a data availability statement. This statement should provide the following information, where applicable:

- Accession codes, unique identifiers, or web links for publicly available datasets
- A list of figures that have associated raw data
- A description of any restrictions on data availability

All data generated or analyzed during this study are included in this published article (and its supplementary and source data information files)

Field-specific reporting

Please select the one below that is the best fit for your research. If you are not sure, read the appropriate sections before making your selection.

☒ Life sciences ☐ Behavioural & social sciences ☐ Ecological, evolutionary & environmental sciences

For a reference copy of the document with all sections, see nature.com/documents/nr-reporting-summary-flat.pdf

Life sciences study design

All studies must disclose on these points even when the disclosure is negative.

Sample size	Sample sizes were chosen based on past experience and typical sample sizes reported in related literature
Data exclusions	In supplementary figure 10, protonemata that didn't show gravitropic bending were considered to be losing gravitropic response due to laser effect and were excluded from the analysis. This is clearly stated in the figure legend.
Replication	All attempts for replication were successful
Randomization	Due to the nature of the experimental setup, randomization was not practical for the experiments reported in this manuscript
Blinding	Due to the nature of the experimental setup, blinding was not practical for the experiments reported in this manuscript

Reporting for specific materials, systems and methods

We require information from authors about some types of materials, experimental systems and methods used in many studies. Here, indicate whether each material, system or method listed is relevant to your study. If you are not sure if a list item applies to your research, read the appropriate section before selecting a response.

Materials & experimental systems

n/a	Involved in the study	
☐	☒	Antibodies
☒	☐	Eukaryotic cell lines
☒	☐	Palaeontology
☒	☐	Animals and other organisms
☒	☐	Human research participants
☒	☐	Clinical data

Methods

n/a	Involved in the study	
☒	☐	ChIP-seq
☒	☐	Flow cytometry
☒	☐	MRI-based neuroimaging

Antibodies

Antibodies used	GFP antibody (Abcam, ab6556, lot GR3252667-1), 1:1000 dilution; FLAG antibody (Sigma, F1804), 1:1000 dilution; anti-Rabbit-AP (cat # 656122, Invitrogen), 1:5000 dilution; anti-mouse-HRP (cat # A9044, Sigma), 1:5000 dilution
Validation	Validation statement can be found at Abcam's documentation (https://www.abcam.com/gfp-antibody-ab6556.html), Sigma's website (https://www.sigmaaldrich.cn/CN/en/product/sigma/f1804?context=product), Invitrogen/Thermo Fisher's website (https://www.fishersci.com/shop/products/anti-rabbit-igg-h-l-ap-conjugated-polyclonal-thermo-scientific-novex-1/656122?searchHijack=true&searchTerm=656122&searchType=RAPID&matchedCatNo=656122), and Sigma's website (https://www.sigmaaldrich.cn/CN/en/search/a9044?focus=products&page=1&perPage=30&sort=relevance&term=A9044&type=product)
